# Evaluation of Virus-Free and Wild-Type Isolates of *Pseudogymnoascus destructans* Using a Porcine Ear Model

**DOI:** 10.1128/msphere.01022-21

**Published:** 2022-03-21

**Authors:** Vaskar Thapa, Nancy P. Keller, Marilyn J. Roossinck

**Affiliations:** a Department of Plant Pathology and Environmental Microbiology, Center for Infectious Disease Dynamics, Pennsylvania State University, University Park, Pennsylvania, USA; b Department of Medical Microbiology and Immunology, Department of Bacteriology, University of Wisconsin, Madison, Wisconsin, USA; University of Georgia

**Keywords:** North American bats, WNS model system, little brown bat, mycovirus, pig ears

## Abstract

White-nose syndrome (WNS), responsible for the mass mortality of North American bats, lacks economically viable and practical *in vitro* models for *Pseudogymnoascus destructans* infection, the causative agent of WNS. Not only are many susceptible North American insectivorous bats nearing extinction and, thus, scarce for experimental studies, but they are difficult to care for and maintain in captivity because of their specialized habitats and diets. In this study, we explored porcine ears as a potential substrate for studying infection development and the dynamics of *P. destructans* growth in the laboratory. Porcine ear skin shares many tissue-level similarities with bat skin and is a readily available resource. We found the porcine ear model provided a substrate faithfully mimicking external *P. destructans* colony morphology and internal histology similar to what is seen with *P. destructans* infections in bat wing membranes. This model provided a major advance by distinguishing virulence attributes between a wild-type *Pseudogymnoascus destructans* strain harboring a partitivirus common to all North American strains of the fungus and an isogenic virus-cured *P. destructans* strain. ImageJ analysis showed that the cured *P. destructans* strain was reduced significantly in ability to produce hyphal cover and showed less spore production on porcine skin. Taken together, these results strengthen our previous finding that the partitivirus infection has a role in WNS and provides a valuable model host tool in understanding *P. destructans* virulence factors for therapeutic application.

**IMPORTANCE** This work describes an important insight into the role of *Pseudogymnoascus destructans* partitivirus in fungal biology and provides a model system for studying white-nose syndrome in bats, which has decimated North American populations.

## INTRODUCTION

*Pseudogymnoascus destructans* is a filamentous, psychrophilic fungus responsible for causing white-nose syndrome (WNS) in North American bats (1, 2). WNS is associated with significant mortality in North American bats since the disease was first reported in 2006 from a hibernaculum in New York state (3). The fungus infects naked bat skin mainly over muzzles, pinnae, patagium (wing membrane), and tail membranes (1). The visual signs of the infection are variable and nonspecific, including roughness, change in skin pigmentation, cracks in the skin, loss of skin gloss, and general lack of inflammation (4). The visual skin lesions are not confirmatory for WNS diagnosis, and histopathologic examinations are required. In wing histology, cup-like epidermal ulcerations filled with *P. destructans* hyphae and conidia are the diagnostic criteria for positive WNS. Fungal invasion deep into the underlying connective tissue, sometimes through the entire width of the membrane, are common signs. On the surface of the membrane, aerial growth of the fungal mycelia is also normally seen (4). On pinnae similar histological features are evident. However, on infected muzzles, the fungal hyphae are commonly found growing in hair follicles and reach up to sebaceous and apocrine glands (4).

Many researchers are working actively to understand the various aspects of WNS, including finding ways to control the disease. All North American isolates of *P. destructans* tested to date are infected with a virus, Pseudogymnoascus destructans partitivirus (PdPV), that has been implicated in some aspects of the fungal biology (5) and has been used as a marker for the epidemiology of the fungus (6). A significant challenge in understanding WNS disease progression is the lack of a good model host. Bats are largely precluded as hosts for several reasons: many bats are nearing extinction; caring for and maintaining bats in captivity for research is difficult because of their special living condition and diets; and bats require regular monitoring for viruses that may have health risks to humans (7). An experimental model system would be a significant tool for many scientific endeavors in various aspects of ongoing WNS research. Whereas prior studies have utilized the insect host Galleria mellonella as a model for *P. destructans* infections (8), there are limitations to insect hosts for mammalian pathogens, especially for dermatophytic fungi. Recently two separate studies evaluated the potential use of porcine skin for studying infection processes of diverse human dermatophytes (9). Porcine skin provides a good model for human skin, especially with reference to porcine ear skin (10).

Given the success of using porcine skin with other dermatophytic fungi, we examined a porcine ear model to study the infection of *P. destructans*. We chose porcine ears because of their similar tissue organization with bat wing membranes and the benefit of easy availability of ears. The bat wing membrane has a tight epidermal layer composed of two tightly packed single layers of cells separated by a layer of connective tissue with elastin fibers. There is a clear demarcation between epidermis and dermis; however, the dermis and hypodermis distinctions are not clear (11). Like the bat wing membrane, porcine ear skin is tightly packed with distinct epidermis and dermis. It is also composed of a larger proportion of dermis, often with indistinct hypodermis (11, 12). We examined the histological characteristics of *P. destructans* infection in porcine ears, and we performed a comparative histological study between the wild-type *P. destructans*, infected naturally with PdPV (5), and nearly isogenic virus-free cultures of *P. destructans* in porcine ears to understand the role of the virus in the biology of *P. destructans* and to validate the method.

## RESULTS

### Virus-free *P. destructans*.

In our previous paper we described curing LBO1 of the virus by treating with cycloheximide, ribaviran, and polyethylene glycol (PEG), producing matric potentials in a range from −2 MPa to −4 MPa in minimal nutrient medium (5). However, after sequence analysis we found that this isolate had about 9,000 single nucleotide polymorphisms (SNPs) compared with the published sequence of North American *P. destructans* (Kurt Lamour, Lightening Genomics, unpublished information). For this study, we did not use any of the drugs that are known mutagens but applied the same PEG method with serial passaging of our wild-type *P. destructans* isolate, LB01, over three generations in PEG at −4 MPa in minimal nutrition media. LB01 and three of these virus-free cultures were sequenced using a next-generation sequencing platform by the laboratory of Kurt Lamour, Lightening Genomics, followed by SNP analysis. SNP analysis showed only a few variations in LB01, LB01-PEG3-1#4, and LB01-PEG3-2#4 (Table 1) compared to *P. destructans* isolate 20631-21 sequence in GenBank (accession no. KV441386 and LAJJ00000000) (13), whereas LB01PEG3-2#4 had more variation. We chose to use LB01-PEG3-1#4 and LB01-PEG3-2#4 as nearly isogenic lines with LB01 for the remaining studies.

**TABLE 1:** SNPs^*a*^

Isolate/culture	SNP type (%)				
	Transition	Transversion	Deletion	Insertion	Total
LB01	0.0067	0.0041	0.0000	0.0000	0.0108
LB01-PEG3-1#4	0.0001	0.0001	0.0000	0.0000	0.0002
LB01-PEG3-2#4	0.0318	0.0101	0.0012	0.0009	0.0440

a SNPs are from virus-infected (LB01) and virus-free cultures of *P. destructans* (LB01-PEG3-1#4 and LB01_PEG3-2#4) and are compared to the published isolate 20631-21 (GenBank accession no. KV441386, BioProject no. PRJNA276926).

### *P. destructans* growth on porcine ear.

**FIG 1:**
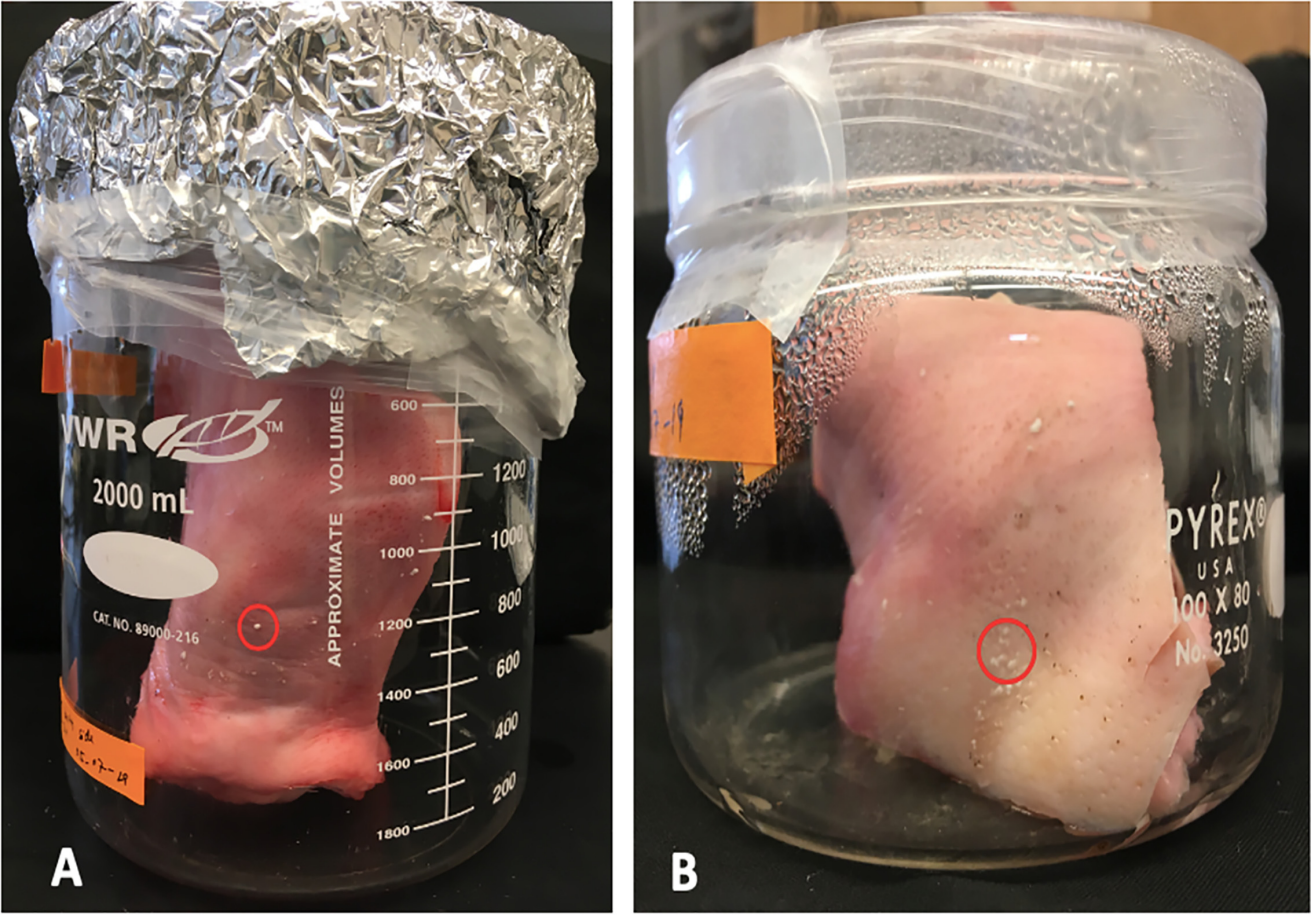
Initial fungal growth on porcine ear surface. Visual signs of *Pseudogymnoascus destructans* (Pd) as a tiny cottony white colony, marked by a red circle, 6 days postinoculation. (A) Intact ear. (B) Cut ear piece. LB01 wild-type isolate of *P. destructans* was used in the experiment.

**FIG 2:**
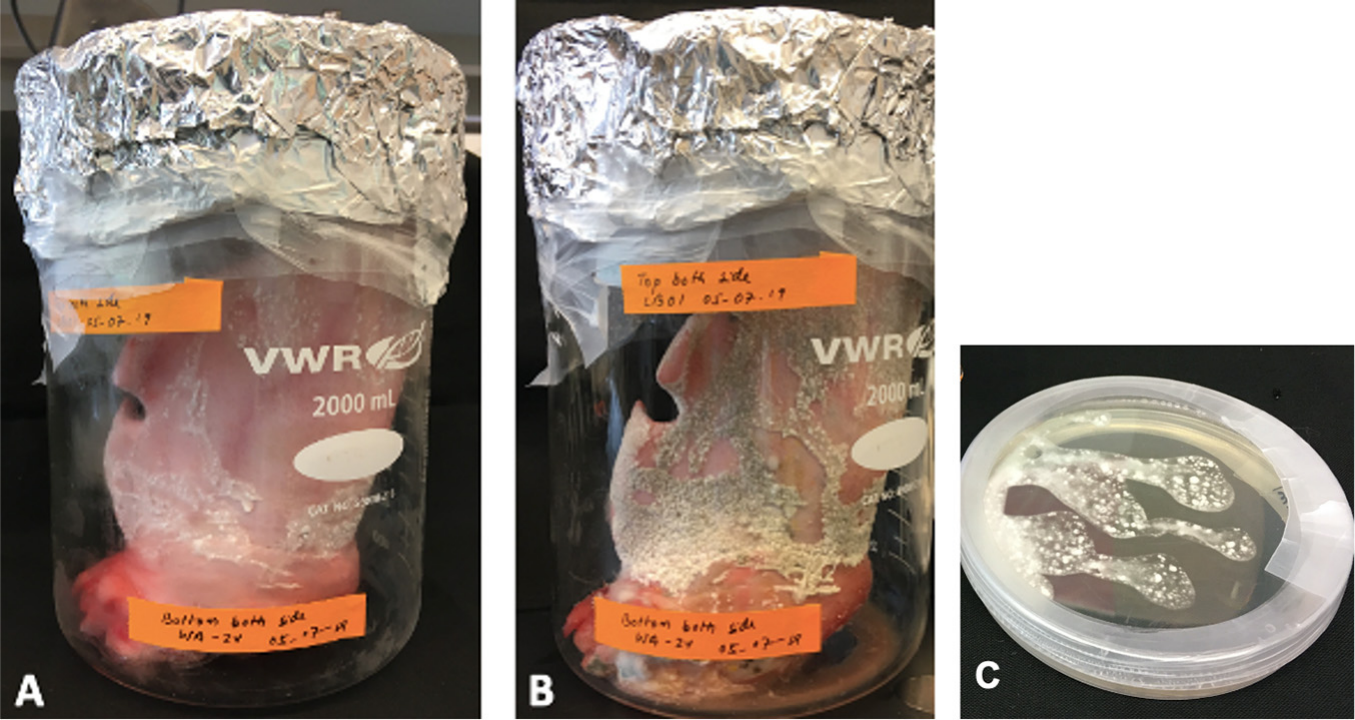
Continued growth of *P. destructans*. Growth of *P. destructans* 2 weeks after inoculation (A) and 3 weeks after inoculation (B) on porcine ear skin surface. (C) SDA plate of *P. destructans* 3 weeks after inoculation. The growth of the fungus progressed over time with colonies appearing gray after 3 weeks due to the formation of conidia.

We observed the first visible sign of LB01 *P. destructans* growth on the porcine ear tissue on the sixth day postinoculation (Fig. 1). The fungus first appeared as tiny round cottony white colonies over the surface of the porcine ear tissue. The growth of *P. destructans* progressed over time and covered almost 40% of the skin surface in 2 weeks (Fig. 2A). In the third week, we observed a gradual change in the fungal colony color to gray due to the production of conidia in the tissue colonized by LB01 (Fig. 2B). We confirmed *P. destructans* growth on porcine ear by PCR using the standard primer pairs for internal transcribed spacer (ITS), elongation factor (EF), and glyceraldehyde-3-phosphate dehydrogenase (gpd) genes on random *P. destructans* colonies growing on the porcine ear surface (Fig. S3).

10.1128/msphere.01022-21.3 FIG S3 Representative profiles of bands amplified using standard primers pairs for the internal transcribed spacer (ITS), elongation factor (EF), and glyceraldehyde-3-phosphate dehydrogenase (gpd) from DNA extracted from the fungal colonies growing on the surface of porcine ears postinoculating spore suspension of LB01 isolate and its virus-free cultures of *Pseudogymnoascus destructans*. M is the marker lane loaded with 1 kb plus DNA ladder from NEB. Download FIG S3, PDF file, 0.9 MB. Copyright © 2022 Thapa et al. 2022 Thapa et al. https://creativecommons.org/licenses/by/4.0/ This content is distributed under the terms of the Creative Commons Attribution 4.0 International license.

### Histology of the wild-type *P. destructans*-infected porcine ear skin.

**FIG 3:**
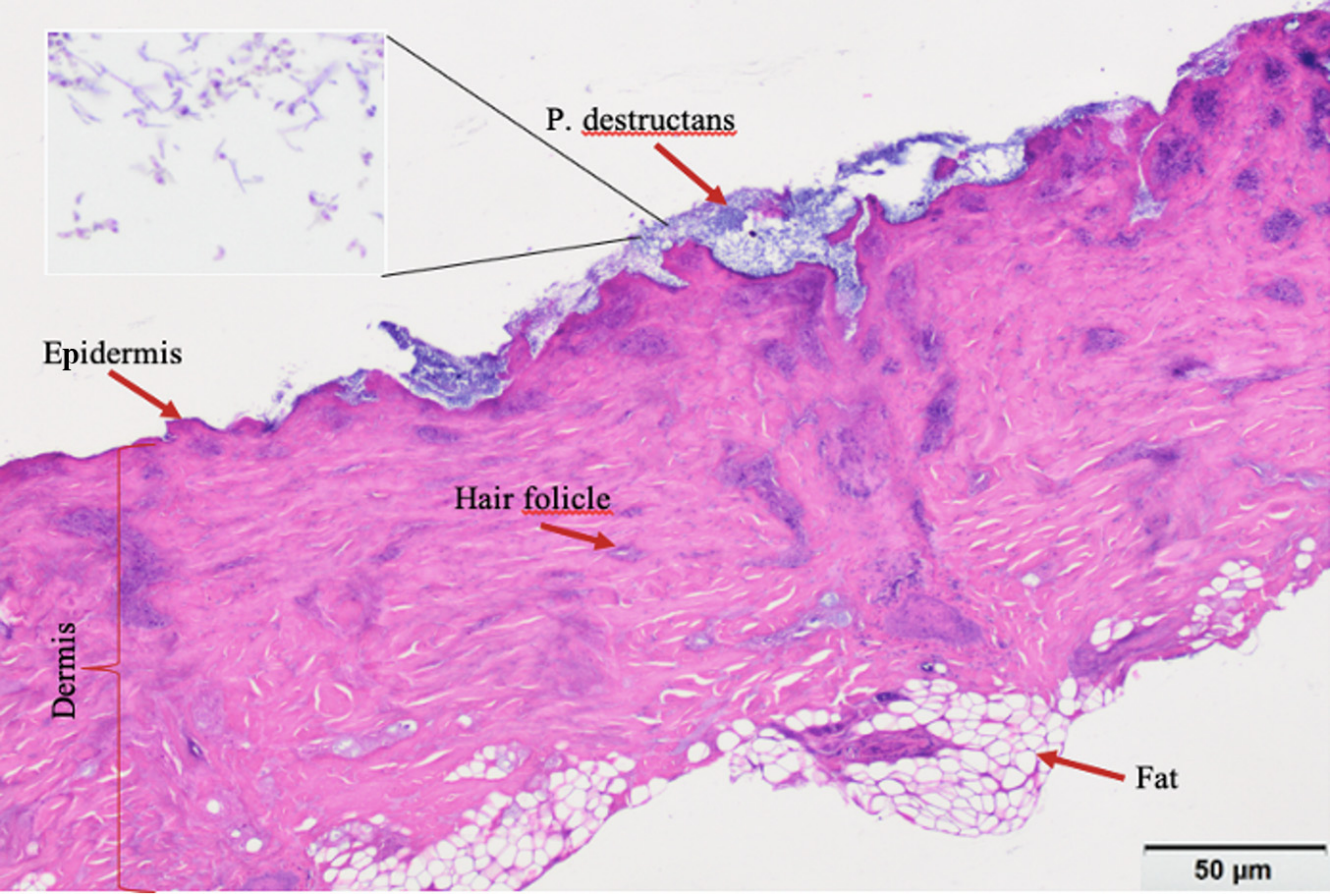
Histology of wild-type *P. destructans* on porcine ears. A 10-μm transverse section of porcine ear stained with hematoxylin and eosin. *P. destructans* can be seen growing over the epidermal layer and rupturing the epidermis with cup-like depressions (marked and labeled).

**FIG 4:**
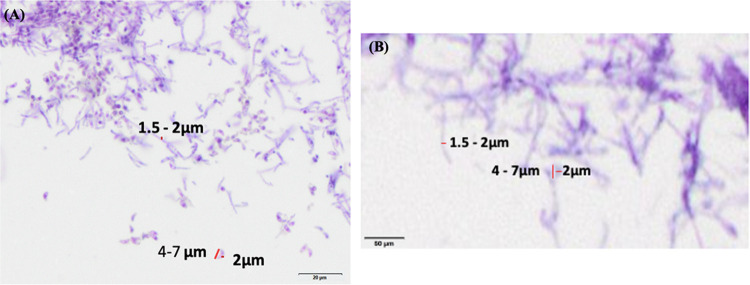
Magnified portion of the *P. destructans* hyphal mass. (A) Wild-type *P. destructans* hyphal mass showing a range of hyphal diameters and range of the width and length of the conidia. (B) Measurement of hyphal diameter and width and length of conidia from virus-free *P. destructans* isolate.

The tissue sections showed LB01 *P. destructans* hyphal mats growing on the epidermal layer of the skin and bearing profuse curved conidia characteristic of *P. destructans*. The epidermal layers were ruptured in many places and the fungus was found growing in cup-like depressions (Fig. 3), similar to *P. destructans* growth patterns noted in infected bat skin (4, 14). The diameter of hyphae was in the range of 1.5 to 2 μm. The conidia were, on average, approximately 2 μm wide and 4 to 7 μm in length on the curved side (Fig. 4A). The measurements were consistent with the findings from the earlier reports (4, 14).

### Histology of the porcine ear skin infected with virus-free *P. destructans* isolates.

**FIG 5:**
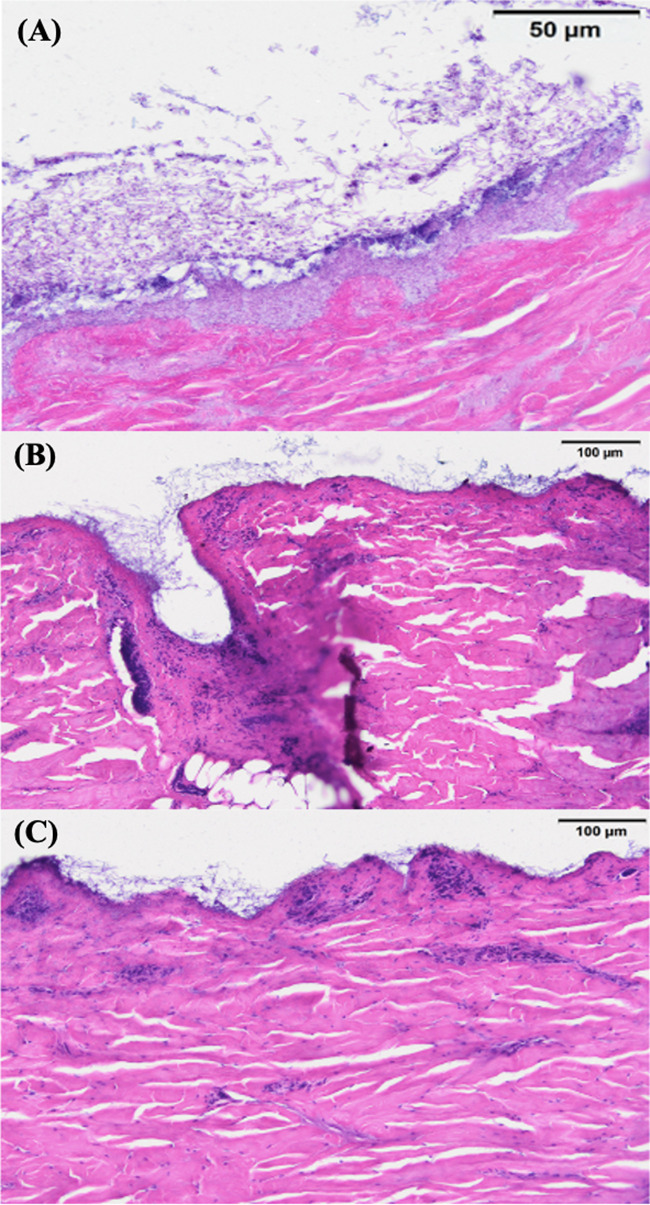
Fungal growth of virus-free *P. destructans*. Visual observation of fungal growth on porcine ears infected with LB01 (A), LB01-PEG3-1#4 (B), and LB01-PEG3-2#4 (C) at 3 weeks postinoculation.

**FIG 6:**
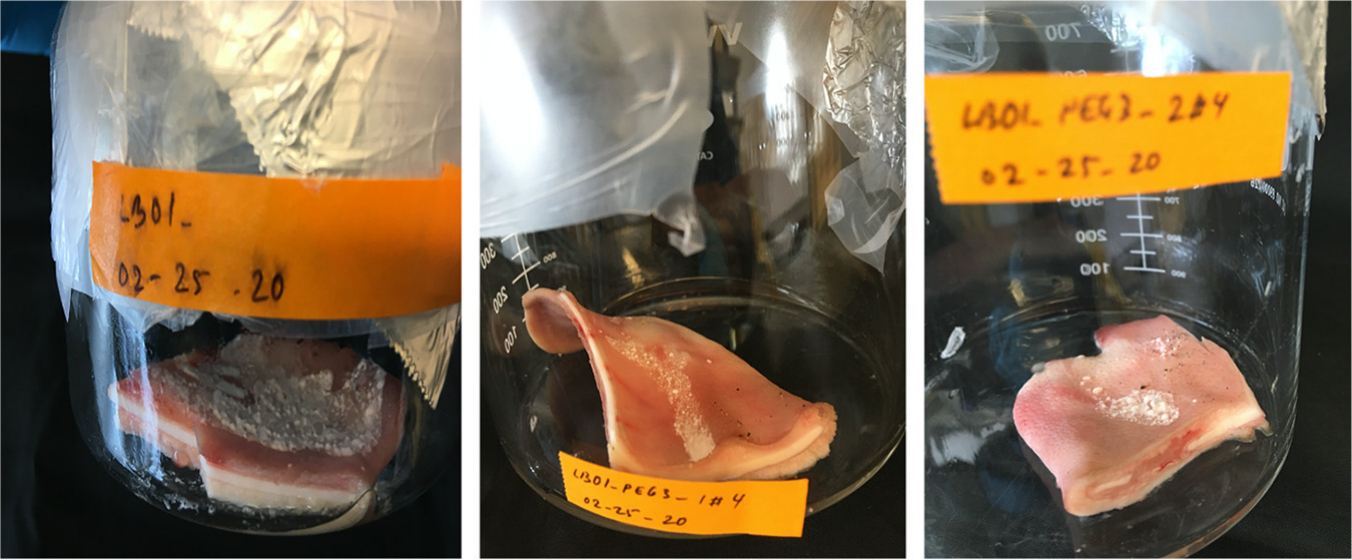
Histology of wild-type versus virus-free *P. destructans*. Comparative histology of porcine ear skin infected with wild-type LB01 isolate of *P. destructans* (A), LB01-PEG3-1#4 (B), and LB01-PEG3-2#4 (C) 3 weeks postinoculation by conidial suspension. All sections were 10-μm transverse sections, stained with hematoxylin and eosin. In sections B and C, infected with virus-free cultures of LB01, no dense fungal growth is seen, although epidermal cup-like depressions are evident (B).

In all tissue sections infected with the virus-free cultures LB01-PEG3-1#4 and LB01-PEG3-2#4, we found *P. destructans* growing loosely on the surface of the porcine ear epidermis. The samples showed the cup-shaped epidermal erosions common with the infection of wild-type *P. destructans* isolates infected naturally with PdPV-pa (Fig. 5A, B, and C). The hyphal mass was less dense for both cured isolates than for the wild-type control *P. destructans* isolate (Fig. 5A, B, and C). Further, the virus-free *P. destructans* colonies maintained white coloration after 3 weeks of growth compared with the grayish color of the wild type, reflecting the reduced production of the fungal conidia (Fig. 6A, B, and C). The hyphal diameter (1.5 to 2 μm) and conidial size (2 μm wide and 4 to 7 μm in length) in the porcine tissue were comparable with the wild-type isolate (Fig. 4B).

### Growth of virus-infected and virus-free *P. destructans* on porcine ear.

**FIG 7:**
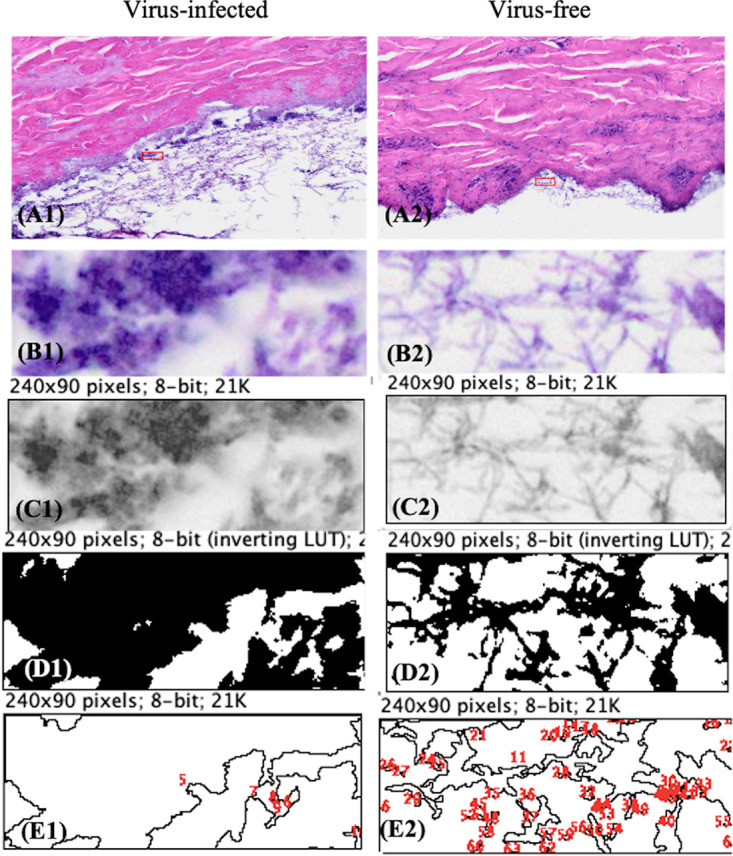
Measurement of hyphal mass as percent cover in ImageJ. Representation of sub-image processing in ImageJ to calculate hyphal cover percentage for virus-infected (A1 to E1) and virus-free *P. destructans* (A2 to E2) growing on porcine ear. Random sampling of 240- by 90-pixel sub-images for analysis, virus-infected (B1) or virus-free (B2). Conversion of selected sub-images to 8-bit gray scale images, virus-infected (C1) virus-free (C2). Adjusting threshold range from 0 to 210 on the 8-bit images for virus infected (D1) and virus free (D2). Numbering polygons to measure areas covered by hyphal mass to calculate percent cover for virus infected (E1) and virus free (E2).

**FIG 8:**
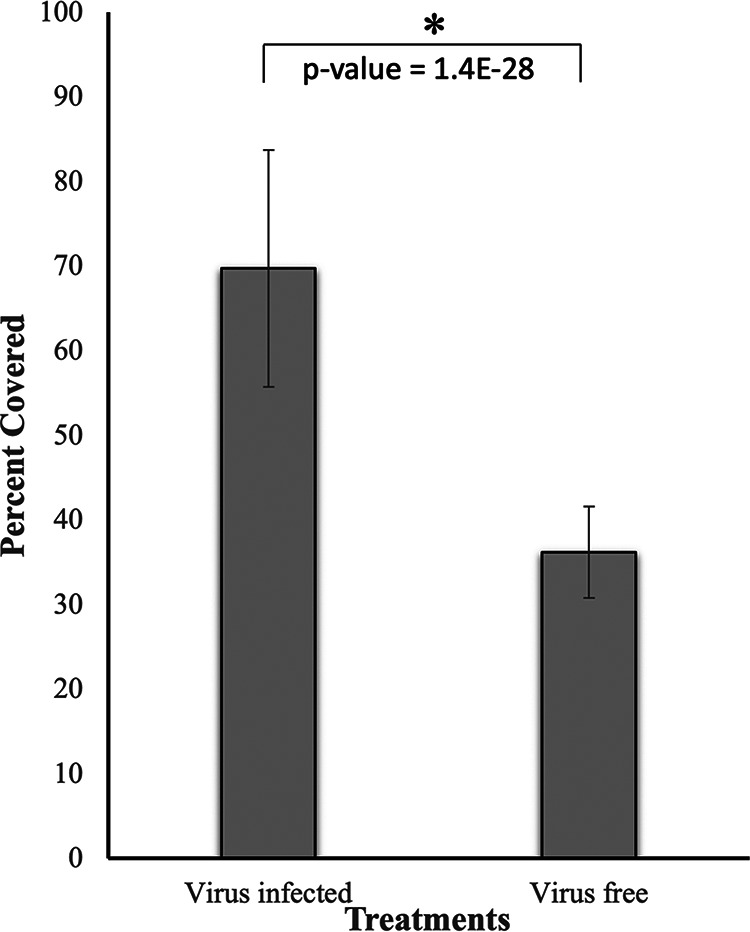
Significant difference in mean percent cover between virus-infected and virus-free treatments. Mean percent cover calculated from 64 random 240- by 90-pixel sub-images from virus-infected (mean, 70 ± 14) and virus-free (mean, 36 ± 5) treatments are significantly different at 95% significance level (*P* = 1.4E−28).

The growth of wild-type *P. destructans* in porcine ear was visually aggressive compared with its growth on Sabouraud dextrose agar (SDA) control plates (Fig. 2B, and C). However, the morphology of hypae on porcine ear and on the plates was similar (Fig. 2A, B, and C). The growth of virus-infected and virus-free *P. destructans* on porcine ear was measured as percent cover of hyphal mass using subsets of images (*n* = 64) in ImageJ (Fig. 7). The analysis determined mean percent cover of 70 ± 14 and 36 ± 5, respectively, for the virus-infected and virus-free treatments (Fig. 8). The mean percent cover for the virus-infected treatments was significantly different from the virus-free *P. destructans* (*P* = 1.4E−28, α = 0.05) (Fig. 8).

## DISCUSSION

In this study, we explored the use of porcine ears as a laboratory model for studying *P. destructans* infection and development. We tested the model using two isogenic virus-free *P. destructans* cultures to evaluate the response of *P. destructans* on the porcine ear. We found the porcine model to mimic infections observed in bat tissues (4, 14); furthermore, this system distinguished growth and potential virulence attributes of the wild-type *P. destructans* isolate with two viral cured isolates. Our findings with the virus-free *P. destructans* infection provided valuable information on *P. destructans* growth and establishment on the skin surface that could be important for understanding virulence factors for therapeutic application.

### Porcine ear as an *ex vivo* model.

North American insectivorous bats susceptible to WNS are not easy to manage in captivity because of their special diet and living habitats (7). Additionally, studies suggest that laboratory colonies of bats fail to behave normally due to aberrant echolocation behaviors in laboratory-maintained bats (15). The study of any disease requires a reliable laboratory model system. We chose to study the porcine ear as a model for *P. destructans* growth and infection dynamics because of its similarity in tissue composition to bat wings (8, 9). Under proper sterilization conditions, we were successful in growing *P. destructans* in porcine ears over 3 weeks either in intact porcine ears or in ear pieces without evidence of contamination by bacteria or other fungi to study the dynamics of *P. destructans* infection. Our results showed that morphology of *P. destructans* growth in porcine ear skin was similar to *P. destructans* growth in bat wing membrane and in SDA plates. *P. destructans* colony color, appearance, and maturation over time with conidial production, where colony color matured from cottony white mycelium to gray conidium-laden fungi in both porcine ear skin and on culture plates. Notably, *P. destructans* colonies growing on the porcine ear skin exhibited denser mycelial growth and expanded faster 3 weeks postinoculation compared with *P. destructans* growth on SDA plates.

The histology of porcine ear skin infected with wild-type *P. destructans* showed a dense hyphal mat on the epidermal surface, with frequent cup-like depressions filled with mycelia similar to *P. destructans* growth in bat wing membrane (4, 14). In many places, *P. destructans* hyphae were found growing in hair follicles similar to bat skin with hair (4). Both the external morphology of *P. destructans* colonies on porcine ear surfaces and the histology of the infected porcine ear skin matched *P. destructans* growth characteristics in bat skin, which provided support that porcine ear could be used as a model in laboratories for *P. destructans* infection.

### Histology of virus-free *P. destructans* infection in porcine ear.

In tissue sections of porcine ear skin infected with virus-free cultures of *P. destructans*, we found different growth characteristics compared with the skin infected with wild-type *P. destructans*. We noticed on the histology images that the virus-free cultures of *P. destructans* developed less mycelium than the wild type, and hyphae were more loosely attached to the epidermis with a few epidermal invasions. The analysis of images in ImageJ showed significantly lower percent cover (*P* = 1.4E−28, α = 0.05) of hyphal mass in the virus-free compared with the virus-infected treatments. In our previous study, we showed that the virus-free isolate spread on the plates at the same rate as the virus-infected isolates (5), and we found the same for these additional virus-free isolates (data not shown). However, it was not possible to quantify the hyphal mass directly due to the nature of the agar gel media. The sparse growth of the virus-free *P. destructans* on the surface of the porcine ear skin with no evidence of the epidermal invasions indicates potential roles of the virus in *P. destructans* growth and in developing virulence. We reported earlier that the virus-free *P. destructans* on SDA plates produced about 4-fold fewer conidia than the wild type and looked white due to the presence of very few conidia (5). We noted no change in the fungal colony coloration after 3 weeks of growth with the virus-free isolates in the porcine ear, implying little conidial production.

In summary, the results support the porcine ear as a good model to understand how *P. destructans* establishes infection on the skin surface and that the porcine ear provides a potential tool in studying the infection dynamics of the virus-free *P. destructans*. The histology of the porcine ear skin infected with the virus-free *P. destructans* confirmed that the virus-free *P. destructans* showed different growth traits and development dynamics over time on dermaphytic tissue. The virus-free *P. destructans* infection in porcine ear could provide a vital platform in identifying virulence factors that could lead to many strategies for therapeutic use to control WNS.

## MATERIALS AND METHODS

### *P. destructans* isolates.

In this study, we used a wild-type isolate of *P. destructans*, LB01, that is naturally infected with the partitivirus PdPV-pa (see Fig. S1 in the supplemental material) and two virus-free cultures of LB01 (LB01-PEG3-1#4 and LB01-PEG3-2#4) (Fig. S1). LB01 was isolated from wing tissue samples of a WNS-infected little brown bat found in the Blossburg mine, Tioga County, Pennsylvania, in 2011. The bat infected with LB01 was found dead in the mine and collected by the Pennsylvania Game Commission survey team. The dead bat showed no other cause of death except infection by the fungus (survey notes and Greg Turner, personal communication). We later found the LB01 isolate in many other dead bats, confirming its pathogenicity. Detailed methods of isolation and culture of the wild-type and virus-free isolates are described in an earlier publication (5).

10.1128/msphere.01022-21.1 FIG S1 Double-stranded RNA profile of *Pseudogymnoascus destructans* isolate LB01 and the cultures, LB01-PEG3-1#4 (1#4), LB01-PEG3-2#4 (2#4), and LB01-PEG3-1#2 (1#2), derived from LB01 after polyethylene glycol (PEG) treatments. The two dsRNA bands in LB01 lanes correspond to dsRNA1 and dsRNA2 of PdPV-pa. Although LB01-PEG3-1#2 is virus free, it shows a higher number of single nucleotide variations; thus, it was not used in this study. Lane M is the lambda DNA marker digested with EcoRI and HindIII. Download FIG S1, PDF file, 0.4 MB. Copyright © 2022 Thapa et al. 2022 Thapa et al. https://creativecommons.org/licenses/by/4.0/ This content is distributed under the terms of the Creative Commons Attribution 4.0 International license.

### Porcine ear collection and *P. destructans* inoculation.

Ears were collected from freshly sacrificed animals collected from the Penn State meat laboratory. Immediately after slaughtering, the animals were scalded in boiling water for hair removal and surface sterilization. The ears were then harvested using clean knives, wearing gloves, and transferred to the laboratory in a sterile plastic bag on ice. All further manipulation was done in a class II, type B2 biological safety hood. The ears were removed from the bag using sterile surgical gloves and placed in a sterile glass container. One of the ears was cut into four pieces, two pieces from the top half and two pieces from the bottom half, using a sterile surgical scalpel blade and sterile surgical scissors (Fig. S2A). The other ear was kept intact (Fig. S2B).

10.1128/msphere.01022-21.2 FIG S2 Porcine ear cut into pieces (A) and the intact porcine ear (B) used in the experiment. Download FIG S2, PDF file, 0.3 MB. Copyright © 2022 Thapa et al. 2022 Thapa et al. https://creativecommons.org/licenses/by/4.0/ This content is distributed under the terms of the Creative Commons Attribution 4.0 International license.

On the surface facing outside the intact ear and on each cut piece, 100 μL of a *P. destructans* conidial suspension of approximately 2.5 × 10^8^ conidia/mL was spread with the help of sterile pipette tips, and the conidia were allowed to settle on the surface by keeping the ear in a position that prevented overflow for about 2 min. The conidial load was determined based on the production of conidia over a 3-week period under similar initial load for the virus-free and wild-type isolate. In the experiment, we used the lowest possible load that resulted in colonization on culture plates for both the virus-free and wild-type isolates. The conidia of the wild-type *P. destructans* that had a natural infection of PdPV-pa (LB01) and the virus-free cultures of *P. destructans* (LB01-PEG3-1#4 and LB01-PEG3-2#4) were collected from 3-week-old Sabouraud dextrose agar (SDA) culture plates grown under conditions described previously (5). In the culture plates, 50 μL of Sabouraud dextrose broth (SDB) was added and swirled to loosen the conidia from the *P. destructans* colonies. Any mycelial fragments mixed with the conidia were removed by passing through a 27-gauge needle. The conidia were then suspended in 50% glycerol and 50% SDB.

The intact porcine ear and the pieces were placed in individual sterile glass containers and closed with a glass lid or an aluminum foil sheet. For controls, 100 μL of *P. destructans* conidial suspension of equal concentration was spread on SDA plates. The containers with the inoculated porcine ear and the pieces were incubated at 10°C in the dark along with the control plates. Each day the inoculated porcine ear and the pieces were visually inspected for signs of fungal growth. Once the fungal colonies were formed, colony PCR was performed using standard primers for the internal transcribed spacer (ITS), elongation factor (EF), and glyceraldehyde-3-phosphate dehydrogenase (gpd) regions, followed by sequence analysis to confirm *P. destructans* (Fig. S3). In every step of the experiment, sterile procedures were strictly followed to avoid any contaminations while handling and transferring the samples.

### Fixation of porcine ear tissue for histology.

After 3 weeks of incubation at 10°C in the dark, sections of about 2 cm by 3 cm of the porcine ear tissue with clear visible signs of *P. destructans* colonies on the surface were cut using surgical gloves, sterile scalpel, and scissors. The section was split open from the middle and cut in half. Each section was placed inside a separate histology cassette cushioned with sponge pads. The tissue-loaded cassette was then placed in a specimen container half-filled with 10% neutral buffered formalin and left for 24 h. After 24 h in formalin, the specimen was transferred to 70% ethanol. The specimen remained in 70% ethanol for at least 12 h before starting the next step.

After treating the specimen in 70% ethanol, further fixing, dehydration, and paraffin infiltrations were done using an automatic tissue processor (Leica TP1020). We used reagent stations with an ethanol series (70%, 85%, 95%, and 100%), histosolve, and paraffin where the tissues were automatically treated over seven and a half hours. The total time allocated was 30 min in each ethanol series, histosolve for 40 min, and 45 min in paraffin. Some of the ethanol series, histosolve, and paraffin treatments were repeated automatically (95%, 100%, ethanol, and paraffin repeated twice, histosolve repeated three times).

The tissue was removed from the cassette and embedded in paraffin using a Leica EG1150C. The tissue was kept upright while embedding in paraffin so that its transverse section was taken. After properly embedding the tissue in the correct orientation in paraffin and cooling it enough to settle the paraffin over the cold plate of the Leica EG1150C, the tissue was sectioned using a Thermo Scientific microtome (Shandon Finesse 325 microtome) into 10-μm sections and floated in a luke-warm water bath. Once any wrinkles in the sections disappeared, they were removed by positioning a slide under each section and gradually lifting it out of the water. The slides were then dried over a slide warmer for about 30 min.

The tissue section was stained with hematoxylin and eosin in an automatic stainer (Leica Autostainer XL). The process took 55 min. The tissue section was then mounted with xylene substitute on the slide. Once the slides dried, the sections were observed under a microscope (Olympus BX60), and photographs were taken.

The tissue fixation, sectioning, and staining were done at the core Microscopy Facility of Huck Institutes of Life Sciences, Pennsylvania State University, University Park, PA.

### Image processing to measure fungal coverage.

The images of porcine ear tissue sections colonized with wild-type *P. destructans* and the isogenic virus-free strains were analyzed by applying ImageJ (16). We used 15 images of the same scale (resolution) for the virus-infected and virus-free treatments for the analysis. In images, 240- by 90-pixel-size sections were selected randomly at 50-μm perpendicular distance from the epidermal layer of the tissue. A total of 32 240- by 90-pixel sections with fungal growth were selected for the virus-infected and virus-free treatments each to quantify percent coverage of the fungal growth. In the ImageJ program, each image was converted to 8-bit grayscale for finer intensity contrast. The images then were adjusted to the threshold value, ranging from 0 to 210. The threshold value allows the pixels within the specified range to be distinct in the binary image for analysis. We selected the threshold range arbitrarily to represent the maximum hyphal network that had the same contrast in both treatments. The percent cover was calculated in ImageJ based on the hyphal coverage in the image, and the mean coverage was tested statistically for difference using *t* test at a 95% confidence level.

### Data availability.

All data reported in this paper are available on request.
