# Supplementary figures and images for: Evaluation of Virus-Free and Wild-Type Isolates of Pseudogymnoascus destructans Using a Porcine Ear Model
Source: mSphere. 2022 Mar 21;7(2):e01022-21. doi: 10.1128/msphere.01022-21 (PMC9044960; doi:10.1128/msphere.01022-21)

**Fig S3**

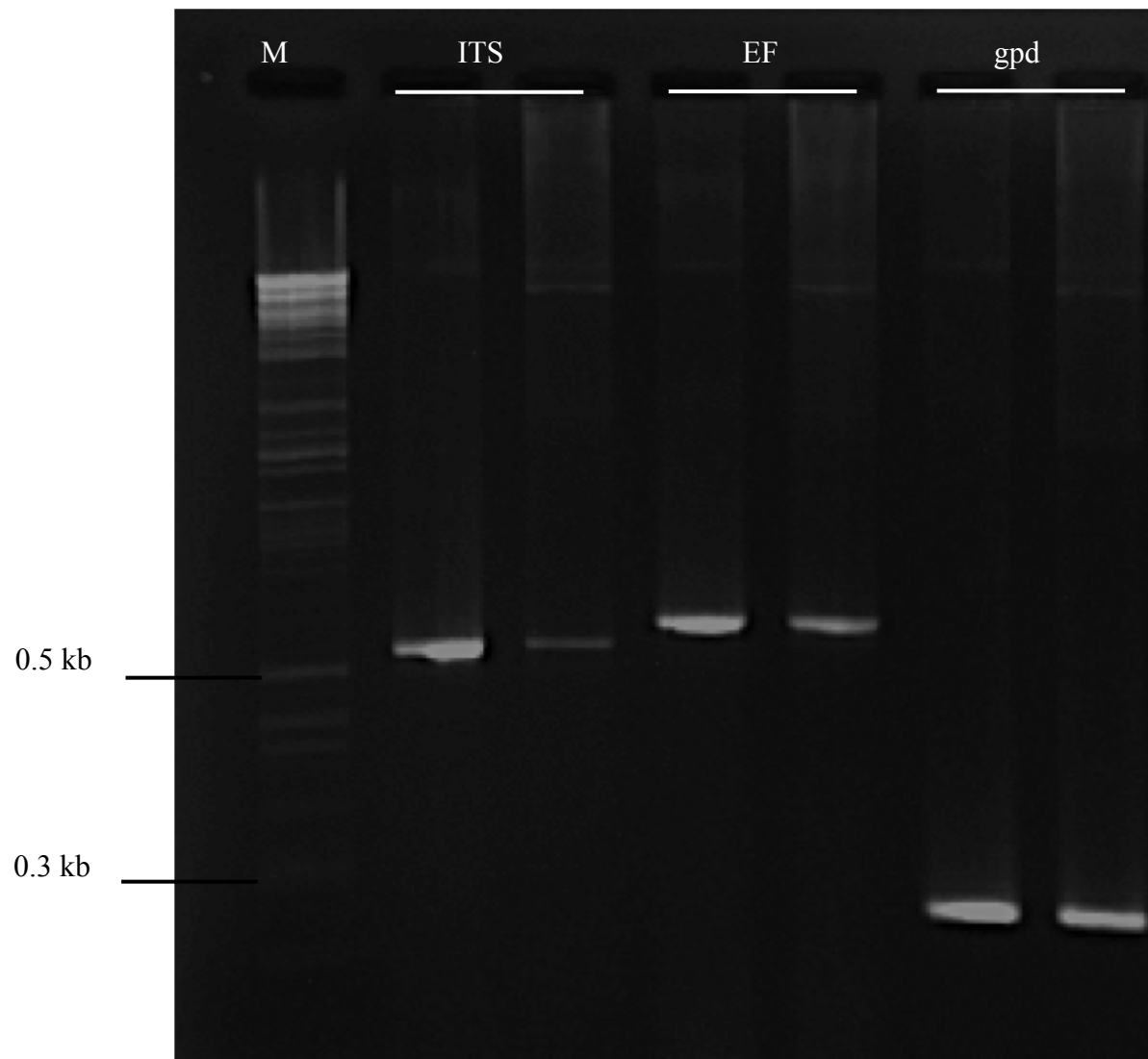

Supplement: FIG S3 [file msphere.01022-21-sf003.pdf]

**Fig S1**

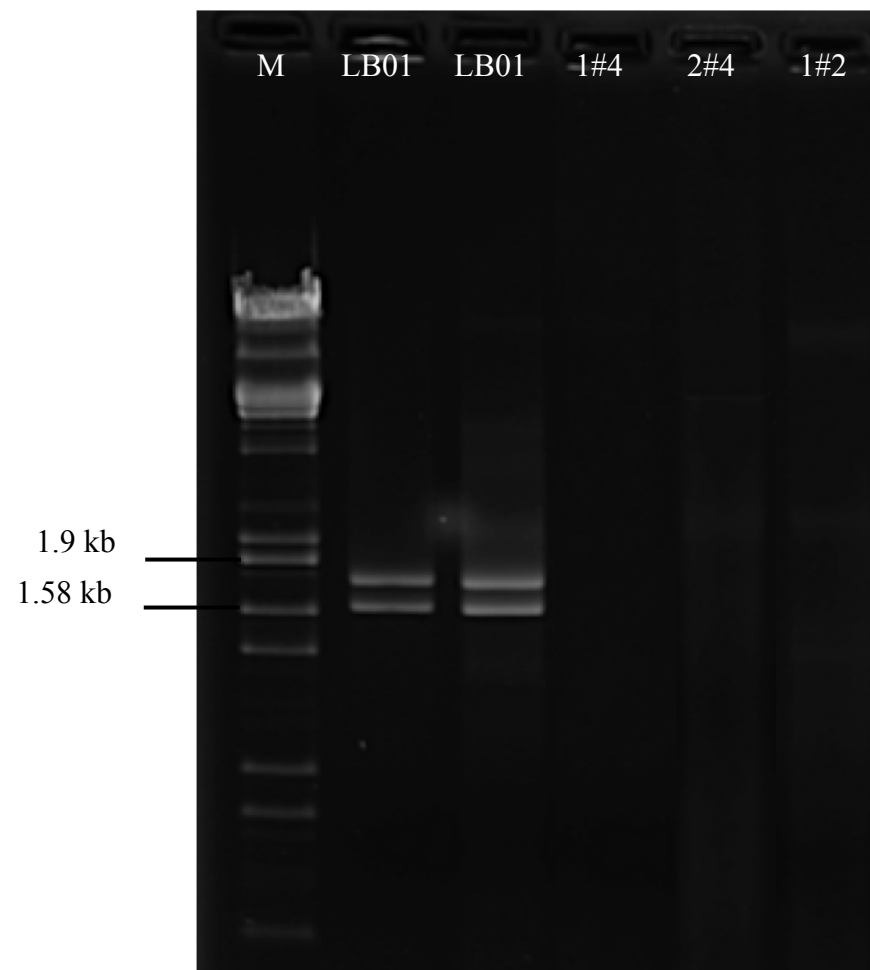

Supplement: FIG S1 [file msphere.01022-21-sf001.pdf]

**Fig S2**

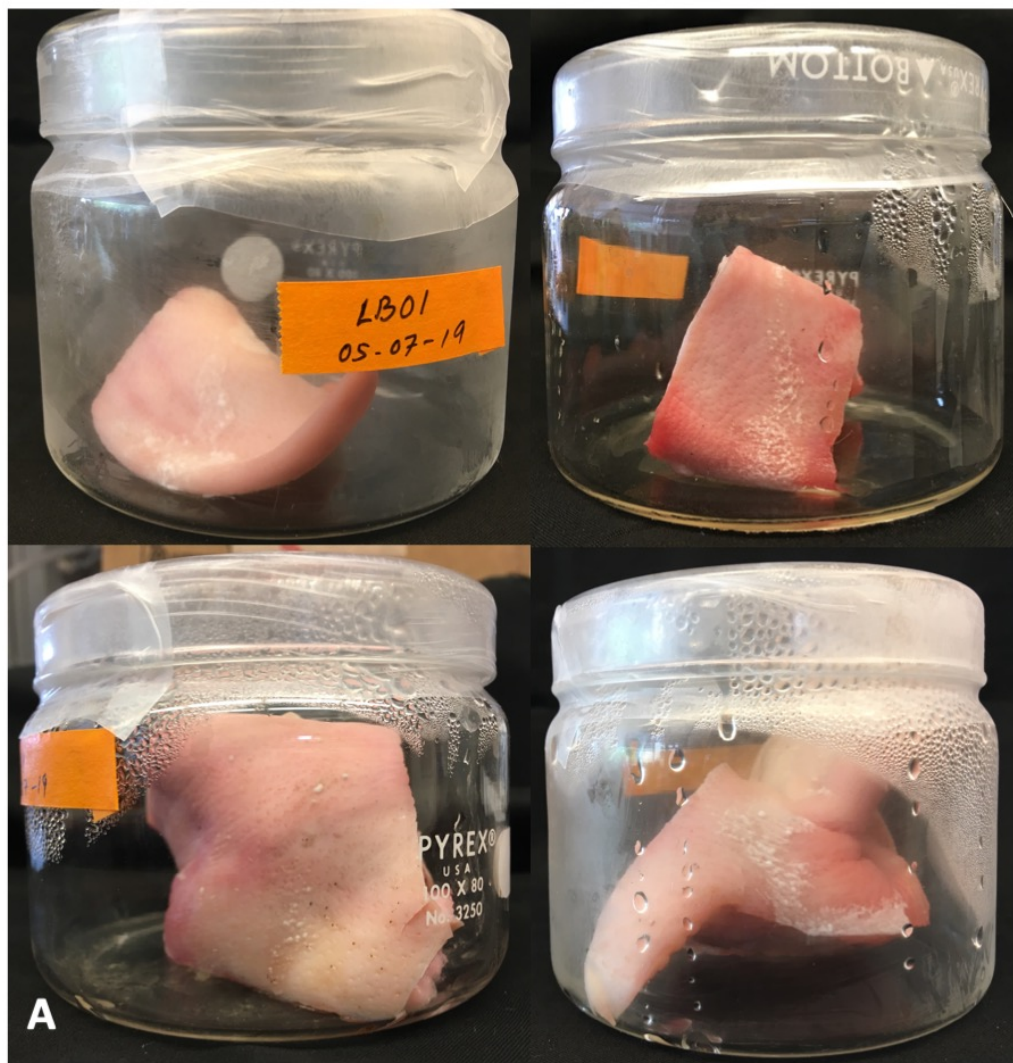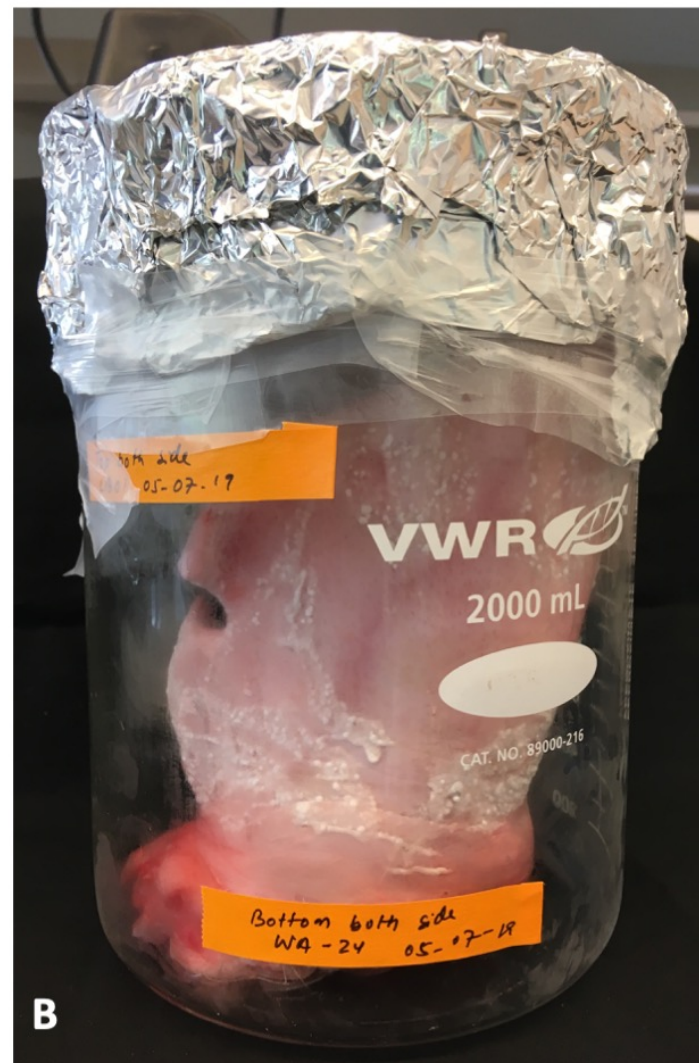

Supplement: FIG S2 [file msphere.01022-21-sf002.pdf]
